# Enhanced Liver Fibrosis Score as a Biomarker for Vascular Damage Assessment in Patients with Takayasu Arteritis—A Pilot Study

**DOI:** 10.3390/jcdd8120187

**Published:** 2021-12-14

**Authors:** Maja Stojanovic, Sanvila Raskovic, Vladimir Milivojevic, Rada Miskovic, Ivan Soldatovic, Sanja Stankovic, Ivan Rankovic, Marija Stankovic Stanojevic, Sanja Dragasevic, Miodrag Krstic, Andreas P. Diamantopoulos

**Affiliations:** 1 Clinic for Allergy and Immunology, University Clinical Center of Serbia, Koste Todorovica 2, 11000 Belgrade, Serbia; sanvilar28@gmail.com (S.R.); rada_delic@hotmail.com (R.M.); 2 Faculty of Medicine, University of Belgrade, 11000 Belgrade, Serbia; dotorevlada@gmail.com (V.M.); doctorranke@gmail.com (I.R.); dragasevicsanja@gmail.com (S.D.); krstic.miodrag61@gmail.com (M.K.); 3 Clinic of Gastroenterohepatology, University Clinical Center of Serbia, 11000 Belgrade, Serbia; 4 Institute of Medical Statistics and Informatic, Faculty of Medicine, University of Belgrade, 11000 Belgrade, Serbia; ivan.soldatovic@med.bg.ac.rs; 5 Center for Medical Biochemistry, University Clinical Center of Serbia, 11000 Belgrade, Serbia; sanjast2013@gmail.com; 6 Faculty of Medical Sciences, University of Kragujevac, 34000 Kragujevac, Serbia; 7 Department of Pathophysiology, Faculty of Medicine, University of Belgrade, 11000 Belgrade, Serbia; marija.stankovic.med@gmail.com; 8 Department of Rheumatology, Akershus University Hospital, 1478 Lørenskog, Norway; adiamanteas@gmail.com

**Keywords:** Takayasu Arteritis, vasculitis, vascular damage, biomarkers, ELF

## Abstract

Takayasu Arteritis (TA) is characterized by granulomatous panarteritis, vessel wall fibrosis, and irreversible vascular impairment. The aim of this study is to explore the usefulness of the Enhanced Liver Fibrosis score (ELF), procollagen-III aminoterminal propeptide (PIIINP), tissue inhibitor of matrix metalloproteinase-1 (TIMP-1), and hyaluronic acid (HA) in assessing vascular damage in TA patients. ELF, PIIINP, TIMP-1, and HA were measured in 24 TA patients, and the results were correlated with the clinical damage indexes (VDI and TADS), an imaging damage score (CARDS), and disease activity scores (NIH and ITAS2010). A mean ELF score 8.42 (±1.12) and values higher than 7.7 (cut-off for liver fibrosis) in 21/24 (87.5%) of patients were detected. The VDI and TADS correlated significantly to ELF (*p* < 0.01). Additionally, a strong association across ELF and CARDS (*p* < 0.0001), PIIINP and CARDS (*p* < 0.001), and HA and CARDS (*p* < 0.001) was observed. No correlations of the tested biomarkers with inflammatory parameters, NIH, and ITAS2010 scores were found. To our knowledge, this is the first study that suggests the association of the serum biomarkers PIIINP, HA, and ELF score with damage but not with disease activity in TA patients. The ELF score and PIIINP may be useful biomarkers reflecting an ongoing fibrotic process and quantifying vascular damage.

## 1. Introduction

Takayasu Arteritis (TA) is a rare, idiopathic, chronic inflammatory disease characterized by granulomatous panarteritis of the aorta and its major branches [1]. Accumulating evidence supports the possibility that matrix metalloproteinases (MMP)-2, MMP-3, and MMP-9 may be sensitive biomarkers for TA activity [2]. The transcriptional expression of MMP-1, -3, -9, and the tissue inhibitor of matrix metalloproteinase-1 (TIMP-1) in peripheral T cells from TA patients has been studied to reveal the influencing factors for activating the adventitial fibroblasts [3]. Furthermore, activated MMP-2/MMP-9 were demonstrated to stimulate myofibroblasts and increase the influx of monocytes/macrophages, leading to oxidative stress, inflammation, and vascular wall injury [4]. Recently, it has been demonstrated that the vasculitogenic T cells migration, through the type IV collagen-containing basement membrane, might be enabled by MMP-9, which is abundantly produced by macrophages [5].

The role of TIMP-1, a natural inhibitor of the proteolytic activity of MMPs, remains controversial in TA. Some studies reported significantly higher levels of TIMP-1 in healthy control individuals than in TA patients [6], while others reported substantially higher levels of TIMP-1 in TA patients than in healthy controls [7]. In 2019, a Chinese group of researchers proposed TIMP-1 as a specific biomarker for TA diagnosis [8].

Liver fibrosis is a dynamic pathological state with an imbalance between MMPs and TIMPs, leading to the deposition of collagen fibers produced by fibroblast-like cells derived from the activated and differentiated hepatic stellate cells [9]. Considering that imbalance between MMPs and TIMPs was seen in chronic liver fibrosis diseases and other diseases characterized by tissue fibrosis, it was hypothesized that the Enhanced Liver Fibrosis score (ELF) might be a suitable marker of overall fibrosis. ELF is a non-invasive test that combines biomarkers directly involved in active fibrogenesis and matrix turnover. It is calculated from three different serum markers: TIMP-1, hyaluronic acid (HA), and aminoterminal propeptide of procollagen type III (PIIINP) [10]. ELF demonstrates the dynamic interplay between fibrogenic and fibrolytic activities, offering excellent diagnostic accuracy in detecting liver fibrosis in chronic liver diseases of different etiology [11].

Although a fibrotic reaction within all three layers of the inflamed aortic wall has been observed, a massively expanded adventitia and typically present intimal fibroplasia may be the main reason for the aortic wall thickness in TA [12]. Keeping in mind that TA is an idiopathic inflammatory disease characterized by vascular fibrosis, some of the local tissue factors that can promote adventitial aortic fibrosis, possibly leading to vessel thickening and remodeling, have recently been identified [13,14].

One of the main challenges in assessing TA is distinguishing damage from disease activity to prevent unnecessary use of cytotoxic medication for newly detected irreversible vascular lesions [15]. Diverse scoring systems have been used to assess damage in TA, such as Vasculitis Damage Index (VDI) and Takayasu Arteritis Damage Score (TADS). These systems encounter irreversible damage in the involved organs, both disease- and treatment-related. VDI and TADS consist of several main categories (11 and 7, respectively), based on clinical findings and do not include imaging data [16,17]. In contrast, Combined Arteritis Damage Score (CARDS) is based on imaging and considers the weight of individual lesions (mild, moderate to severe stenosis, occlusion, and aneurysm) in 25 arterial regions [18]. No biomarkers correlating with vascular damage in TA have been yet explored.

This study aims to investigate the hypothesis that quantitative values of ELF and its constituents, TIMP-1, PIIINP, and HA, may demonstrate fibrosis of blood vessels and serve as markers for disease extent and damage in TA patients.

## 2. Materials and Methods

### 2.1. Patients

This monocentric, cross-sectional study included TA patients who have been followed up with in outpatient and in-patient clinic from February 2018 to February 2019, at the Clinic of Allergy and Immunology, University Clinical Center of Serbia in Belgrade. All the patients met three or more out of six 1990 American College of Rheumatology (ACR) classification criteria [19]. A blood sample for PIIINP, TIMP-1, and HA analysis to perform the ELF calculation was taken from all TA patients who agreed to participate in the study and provided their informed consent. Blood samples for a broad set of biochemical analyses were collected at the time of ELF sampling. Data on age at symptoms onset and diagnosis, follow-up duration, gender, initial vascular involvement, type of vascular lesions, and angiographic type of the disease [20] were retrospectively collected from the patient’s records. The participants’ self-reported smoking history and alcohol intake and use of oral glucocorticoids (converted to the equivalent prednisone dose) and immunosuppressants were assessed retrospectively.

Arterial damage, defined as stenosis, occlusion, or aneurysm, was based on imaging with computed tomography angiography (CTA) and Doppler ultrasound (US) examinations. CTA was not performed routinely, and not if it was not clinically indicated at the last follow-up. To assess the arterial damage of the descending aorta, abdominal aorta, celiac artery, superior mesenteric artery, and renal artery, we used the last CTA performed. US of the ascending aorta, aortic arch brachiocephalic trunk (BCT), carotid, vertebral, subclavian, axillary, and iliac arteries were performed within two months of the blood sampling, by the same vascular radiologist. Arterial damage of the pulmonary and coronary arteries was included if detected on the CTA performed for symptomatic reasons. Arterial involvement on CTA and/or US was evaluated in the following arterial territories for each patient: (1) ascending aorta and aortic arch, (2) carotid and BCT, (3) subclavian, (4) auxiliary, (5) thoracic aorta, (6) abdominal aorta with branches, and (7) iliac artery. According to the arterial involvement, we counted vascular regions as follows: one to five and more than five. Heart and abdominal US were routinely performed in all patients. A non-invasive estimation of the right ventricular mean pressure (RVMP) by Doppler ultrasound in patients with a registered tricuspid regurgitation was performed.

### 2.2. Blood Samples and ELF Analysis

Blood samples were collected into vacutainer tubes (BD Vacutainer Systems, Franklin Lakes, NJ, USA), allowed to clot for 30 min at room temperature, and centrifuged at 1600× *g* for 15 min at 4 °C. Serum was separated into aliquots and frozen at −70 °C until the analysis. The levels of PIIINP, HA, and TIMP-1 were analyzed by the ADVIACentaur^®^immunoassay system (ADVIA CentaurTM, SiemensHealthcare Diagnostics, Tarrytown, NY, USA). The ELF score was auto-calculated by the instrument employing the recommended equation (ELF = 2.494 + 0.846 ln(C_HA_) + 0.735 ln(C_PIIINP_) + 0.391 ln(C_TIMP-1_)) and expressed as a numerical value with no units. The cut-off values of ELF test were applied in line with the manufacturer’s thresholds, validated in large cohorts (<7.7 = no or mild fibrosis; ≥7.7 to <9.8 = moderate fibrosis; ≥9.8 to <11.3 severe fibrosis/cirrhosis; ≥11.3 = cirrhosis) [21].

### 2.3. Damage and Activity Assessment

The damage assessment was performed by calculating the TADS and VDI at the last follow-up. VDI encounters any organ damage since the onset of systemic vasculitis (maximum score 63). On the other hand, TADS is calculated by marking the basic characteristics present at least 6 months (maximum baseline score 29), with a possibility of including more items (each one can be scored with an additional point) to delineate the anatomic site of vascular events, and/or repeated vascular interventions, and/or other damage items [16,17]. CARDS, which can be scored as 40, as a maximum value, was calculated after completing imaging procedures in all patients [18]. The National Institutes of Health (NIH) score [22] and Indian Takayasu Clinical Activity Score (ITAS2010) [23] were used to assess disease activity.

### 2.4. Statistical Analysis

Data were analyzed using the Statistical Package for the Social Sciences 20.0 (SPSS, Chicago, IL, USA). For categorical variables, absolute and relative (%) frequencies were presented; for quantitative variables, mean and standard deviation (SD) or median and interquartile range (IQR) were reported. The Student’s *t* test was used to determine statistically significant difference between two unrelated groups. The Pearson correlation test was used for correlation analysis. A correlation between CARDS and ELF adjusted for age is performed using partial correlation analysis. *p*-value < 0.05 was retained for statistical significance.

## 3. Results

Twenty-four patients were recruited in this study: 22 women (91.7%) and two men (8.3%); their characteristics are summarized in Table 1.

The mean age of appearance of the first symptoms was 40.5 years. The average diagnostic delays and the follow-up durations were 4.29 and 4.9 years, respectively. At the last follow-up, 19 (79%) patients were treated with corticosteroids (mean dose 9.58 ± 6.41 mg, per day), 5 (20.8%) methotrexate, 2 (8.3%) azathioprine, and 2 (8.3%) mycophenolate mofetil. Eleven (45.8%) of TA patients had Type V of the disease, and the same number of patients had the affection of 4 or more vascular regions. The main laboratory findings and clinical characteristics reflecting liver function are presented in Table 2.

No abnormal findings on liver function tests and other biochemical markers were detected. The diagnostic workup for hepatitis B and C, and the human immunodeficiency virus, anti-mitochondrial and anti-liver-kidney microsome type-1 antibodies was negative in all patients. Anti-nuclear antibodies were low positive in seven and anti-smooth muscle antibodies in three patients. Two patients had a concomitant inflammatory bowel disease (one diagnosed with ulcerative colitis and one with Crohn’s disease), and another two had chronic autoimmune thyroiditis. No chronic liver diseases were observed and liver ultrasound was normal except in one patient with non-homogeneous liver and normal laboratory tests, and in two non-obese patients with a hyperechoic liver, without hypercholesterolemia and normal BMI values. No patient self-reported excessive alcohol intake.

Based on NIH activity scoring system, 16 (66.7%) patients had active disease (score ≥2), and eight (33.3%) were in remission. A median of 5.5 (2–15) for ITAS2010 was calculated.

Damage assessment in all patients was calculated by using the VDI, TADS, and CARDS (Table 3).

The ELF score ranged from 6.10 to 10.73, with a mean of 8.42 (±1.12). ELF test values higher than 7.7 were measured in 21 (87.5%) patients: 18 had values between 7.7 and 9.8, while three patients had ELF values between 9.8–11.3. Patients on ongoing MTX treatment had a mean value of ELF 8.29 ± 1.65, while patients not treated by MTX had 8.46 ± 0.99. Statistically significant difference of the biomarkers ELF, TIMP-1, PIIINP, and HA, as well as the damage scores CARDS, VDI, and TADS, between the groups of patients on ongoing/without corticosteroid treatment at the assessment, was not determined.

The correlation between the ELF score and each of the three markers (TIMP-1, HA, PIIINP) with damage indexes and clinical characteristics is presented in Table 4.

The correlation between the VDI and TADS, and ELF value was significant (*p* < 0.01). Additionally, a robust correlation between ELF and CARDS (*p* < 0.0001) was observed. Due to a high correlation between age and ELF value (r = 0.51; *p* = 0.01), as well as age and CARDS (r = 0.43; *p* = 0.03), at the next step, a partial correlation, which was an age-adjusted analysis for ELF and CARDS, was performed, showing a high, positive, and statistically significant correlation (r = 0.730; *p* < 0.001).

No correlations of the biomarkers (ELF, TIMP-1, PIIINP, and HA) with the ESR, CRP level, disease duration, and activity scores (ITAS2010) were observed. A mean ELF value of 8.4 ±1.24 was measured in NIH active, while NIH non-active TA patients had a mean ELF 8.46 ± 0.92. No statistical significance was found between the two groups (*p* = 0.903). The ELF score and each biomarker itself were found to correlate with the number of vascular regions involved (*p* < 0.05 for HA concentration, and *p* < 0.01 for the rest). The ELF score and the concentration of HA correlated with age: *p* < 0.01, *p* < 0.05, respectively.

## 4. Discussion

To our knowledge, this is the first study that evaluates the profile of extracellular matrix biomarkers and suggests their possible positive correlation with damage indexes in TA patients. Although the ELF score has not been previously examined in TA patients, its constituents, TIMP-1 and PIIINP, were studied mainly as diagnostic or TA activity biomarkers.

Interestingly, significantly elevated serum concentrations of HA, PIIINP, and TIMP-1, and ELF score, in patients with IgG4-related disease comparing to healthy controls, were previously reported [24]. Furthermore, in 2019, ELF was validated as a useful fibrosis marker in systemic sclerosis (SSc). Likewise, the ELF score’s value as an independent marker of disease severity, skin, and lung involvement, was confirmed in this multicentric study that included 457 patients with SSc [25].

As previously mentioned, TIMP-1 has recently been proposed as a specific diagnostic marker for TA. In line with this, only five (20.83%) of the patients from our cohort were identified to have more than 221.86 μg/L, which is suggested as a cut-off value [8]. TIMP-1 is a natural inhibitor of MMPs, playing an essential role in regulating the ECM turnover and affecting the proliferation, migration, and apoptosis of vascular cells [26]. While the precise mechanism remains elusive, the transforming growth factor (TGF)-β pathway may be crucial for TA’s vascular fibrosis [27]. It is interesting to note that the release and activation of TGF-β within ECM controlled by MMPs increases ECM deposition by stromal cells and fibrosis and may be inhibited by TIMPs [28]. Some studies have reported that the serum levels of TGF-β follow TA activity [29,30,31]. The differentiation of fibroblasts into myofibroblasts, regulated by the ECM molecules and TGF-β by stimulating α-smooth muscle actin (α-SMA), leads to higher collagen production within vessel walls [32,33]. Significantly higher collagen I, collagen III, fibronectin, α-SMA, TGF-β in TA arteries was found compared to the normal arteries [34]. Consequently, lower TIMP-1 levels in our TA patients may suggest the possibility of a TIMPs/MMPs imbalance, leading to a higher profibrotic activity of TGF-β and thus to vascular impairment.

Dexamethasone has proven to downregulate TIMP-1 in vitro [35]. The ongoing corticosteroid treatment in 79% of patients at the last visit may additionally explain the lower concentrations of TIMP-1 in our TA cohort.

In a recent study, TIMP-1 was identified as a potential marker of active giant cell arteritis (GCA). At the same time, no significant differences in the TIMP-1 concentrations were detected in TA, polyarteritis nodosa (PAN), and eosinophilic granulomatosis with polyangiitis (EGPA), both in active disease and remission [36]. On the other hand, the MMP-9, TIMP concentrations, and the MMP-9:TIMP ratio were diminished in patients with Kawasaki disease (KD) [37]. Clinical and etiopathogenetic heterogeneity between other vasculitides, treatment with corticosteroids and other immunosuppressives, and disease variability between patients with the same form of vasculitis may explain the different results of diverse studies.

Unlike TIMP-1, PIIINP was significantly associated with TA damage indexes VDI and TADS, although the strongest correlation existed across PIIINP and CARDS. This agrees with the previous finding that PIIINP remains increased in young adults with KD, especially in patients with persistent coronary artery lesions (CAL). Moreover, concentrations of PIIINP were closely associated with quantifiable characteristics of the severity of coronary stenosis/occlusion and coronary thrombosis (CAL) in KD patients [37]. In addition, PIIINP correlated with particular echocardiographic parameters in patients with rheumatic heart diseases and the right ventricular deterioration due to myocardial fibrosis [38,39]. In addition, serum concentrations of PIIINP and TGF-β were associated with peripheral artery disease involving large vessels and findings of a stiffer aorta in patients with dilated cardiomyopathy [40,41]. Thus, we assume that PIIINP may be involved in the process of vascular fibrosis in patients with TA, explaining the strongest correlation with CARDS rather than other damage indexes (VDI, TADS). This fact also suggests a possible linear correlation between quantitative serum PIIINP levels and disease progression depended on a fibrotic process within the vessel wall, similar to those reported in patients with KD [37].

Our present findings revealed the association of the serum concentration of HA with all damage indexes, mostly to CARDS. HA is an essential component of the extracellular matrix in virtually every body tissue, including the vasculature [42]. HA accumulates in the artery wall in response to injury and inflammation within the atherosclerotic intima and fibroproliferative lesions in the balloon-injured rat carotid arteries [43,44]. The role of HA in TGF-β-driven myofibroblast activation has recently been reported [45]. Interestingly, Lymphatic Vessel Endothelial Hyaluronan Receptor 1 (LYVE-1), a protein capable of HA binding and clearance, was shown to be dependent on inflammatory cytokines, and the tumor necrosis factor (TNF)-α induced a down-modulation of LYVE-1 in ex vivo murine dermal tissue explants [46,47]. Recently, LYVE-1 was identified as a potential biomarker of TA activity [8]. Consequently, arterial injury followed by tissue remodeling in TA might influence both LYVE-1 expression and serum concentration HA in TA patients.

For the first time, the present study identified PIIINP, HA, and ELF as biomarkers associated to damage, but not with activity in TA patients. Damage denotes chronic disease aspects that are not likely to respond to immunosuppressive therapy and significantly influence both long-term prognosis and quality of life [48]. In daily clinical practice, physicians are often prompted to escalate and change therapies based on newly identified vascular progression (i.e., new stenosis) rather than the measurement of disease activity and damage assessment [49]. VDI was designed to calculate overall damage in any vasculitic syndrome, including large-vessel vasculitides [16], while TADS was more adjusted to TA, paying more attention to the items related to the cardiovascular system [17]. An older age at symptom-onset and disease duration predicted TADS≥8, whereas age at symptoms and cumulative CS dose were independent factors for VDI≥5 [47]. Many patients may have already encountered significant amounts of damage at the time of enrolment in clinical trials, and thus it may also be essential to specify the level of baseline damage [48].

On the other hand, CARDS represents numerical vascular damage based only on imaging findings not correlated with activity biomarkers [18]. This may partly explain why the ELF values, which may quantitatively reflect an overall fibrotic process in TA, were in the strongest correlation with CARDS, suggesting its possible role in the estimation of disease extent, rather than representing all-cause damage measured by VDI and TADS. Accordingly, an accurate characterization of the vascular damage at baseline and along the disease course might be critical for therapeutic decisions, follow-up, and prediction of clinical outcomes, and also may influence the therapeutic strategy in the direction of the development of new anti-fibrotic drugs.

This study’s main limitations are the small sample size and the lack of validation of ELF cut-off values for TA patients. Periodical damage assessment and biomarkers measuring along the disease course in a longitudinal study design would more precisely define the role of ELF score in clinical practice and clinical trials.

## 5. Conclusions

In conclusion, serum PIIINP, HA, and ELF score may be useful biomarkers reflecting an ongoing fibrotic process and quantifying vascular damage in TA patients. These encouraging results warrant further investigation in larger TA patient cohorts.

## Figures and Tables

**Table 1:** Demographic and clinical patients’ characteristics.

General		
Age at symptoms onset (years), mean ± sd	40.5 ± 15.5	
Age at diagnosis (years), mean ± sd	44.8 ± 15.4	
Delay at diagnosis (years), med (IQR)	2.5 (1–4.75)	
Age at lab recruitment (years), mean ± sd	49.15 ± 16.0	
Follow up duration (years), med (IQR)	3.5 (1–6.0)	
Treatment (Present)	*N* (%)	Duration (months), Range
CS	19 (79%)	32.75 (0–240)
MTX	5 (20.8%)	7.58 (0–120)
AZA	2 (8.3%)	9.38 (0–125)
MMF	2 (8.3%)	5.5 (0–96)
No treatment	4 (16.7%)	
Angiographic Type	*N* (%)	
I	1 (4.2%)	
II	9 (37.5%)	
III	2 (8.3%)	
IV	1 (4.2%)	
V	11 (45.8%)	
Number of Vascular Regions Involved	*N* (%)	
1	2 (8.3%)	
2	5 (20.8%)	
3	6 (25.5%)	
4	5 (20.8%)	
5	2 (8.3%)	
>5	4 (16.6)	
Morphological Type	*N* (%)	
Stenotic	13 (54.2%)	
Aneurysmal	5 (20.8%)	
Combined	5 (20.8%)	
Wall thickness	1 (2.4%)	

CS: corticosteroids; MTX: methotrexate; AZA: azathioprine; MMF: mycophenolate mofetil.

**Table 2:** Laboratory parameters, diagnostics, and other patients’ characteristics.

Laboratory	
CRP mg/L, med (IQR)	13.05 (4.45–22.95)
Plt 10^9^/L, mean ± sd	266.6 ± 88.4
AST U/L, med (IQR)	19.5 (14.25–26.75)
ALT U/L, med (IQR)	21.5 (14.0–27.5)
AP U/L, med (IQR)	62.5 (44.75–79.75)
GGT U/L, med (IQR)	23.0 (14.0–41.5)
Bilirubine umol/L, med (IQR)	6.65 (5.65–11.22)
Albumine g/L, mean ± sd	37.85 ± 4.74
Total proteins g/L, mean ± sd	69.42 ± 6.43
IgG g/L, mean ± sd	11.15 ± 3.77
IgM g/L, mean ± sd	1.24 (0.58–2.61)
Chol total, mmol/L mean ± sd	5.07 ± 0.88
Tg mmol/L, mean ± sd	1.47 ± 0.73
**Liver US**	***N* (%)**
Normal	21 (87.5%)
Hyperecholic	2 (8.3%)
Non-homogeneous	1 (4.2%)
**Other Characteristics**	
BMI (kg/m2), mean ± sd	24.32 ± 4.03
RVMP (mmHg), mean ± sd	28.71 ± 6.26
**Smoking**	***N* (%)**
Never	12 (50%)
Past	5 (20.8%)
Present	7 (29.2%)

CRP: C-reactive protein, Plt: platelets, AST: aspartate aminotransferase, ALT: alanine aminotransferase, AP: alkaline phosphatase, GGT: gamma glutamyl transferase, IgG: immunoglobulin G, IgM: immunoglobulin M, US: ultrasound; BMI: body-mass index; RVMP: right ventricle mean pressure.

**Table 3:** Assessment of Damage in TA patients.

Damage Score	
VDI med, range	4.00 (0–10)
TADS med, range	5.00 (0–10)
CARDS med, range	9.10 (2–14)

VDI: Vasculitis Damage Index; TADS: Takayasu Arteritis Damage Score; CARDS: Combined Arteritis Damage Score.

**Table 4:** Correlation coefficient (r) between ELF, TIMP-1, PIIINP, HA and clinical characteristics, damage and activity scores.

	ELF	TIMP-1 (ng/mL)	PIIINP (ng/mL)	HA (ng/mL)
Results	8.42 ± 1.12	207.8 ± 81.2	7.64 ± 3.00	17.51 (11.88–36.7)
Age	0.51 **	0.31	0.34	0.45 *
Disease duration	0.18	0.03	0.17	0.27
ESR	−0.06	−0.15	−0.29	−0.17
CRP	−0.11	0.02	−0.13	0.06
ITAS2010	0.65	0.19	−0.09	0.19
VDI	0.54 **	0.38	0.51*	0.50 *
TADS	0.59 **	0.28	0.53 *	0.47 *
CARDS	0.79 ****	0.39	0.69 ***	0.66 ***
Number of vascular regions involved	0.59 **	0.58 **	0.55 *	0.58 **

ELF: Enhanced Liver Fibrosis score, TIMP-1: tissue inhibitor of matrix metalloproteinase-1, PIIINP: procollagen-III aminoterminal propeptide, HA: hyaluronic acid, ESR: erythrocyte sedimentation rate, CRP: C-reactive protein, ITAS2010: Indian Takayasu Arteritis Activity Score; VDI: Vasculitis Damage Index; TADS: Takayasu Arteritis Damage Score; CARDS: Combined Arteritis Damage Score; Level of significance: * *p* < 0.05; ** *p* < 0.01; *** *p* < 0.001; **** *p* < 0.0001.

## Data Availability

The data presented in this study are available upon a reasonable request, from the corresponding author.
